# Supplementary material for: Content-rich biological network constructed by mining PubMed abstracts
Source: BMC Bioinformatics. 2004 Oct 8;5:147. doi: 10.1186/1471-2105-5-147 (PMC528731; doi:10.1186/1471-2105-5-147)
Supplement: Additional File 2 — The original results of the above study (non-essential files are deleted to keep the file size under the limit set by BMC bioinformatics). [file 1471-2105-5-147-S2.bz2 › chilibotAdditionalFile2/dip05/341ID8076595E4256/html/TNFSF4_TNFRSF4.html]

 


 **TNFSF4** and **TNFRSF4** 
  
Found 114 abstracts in PubMed, retrieved 05.  
 

 What does Google say? 
 PDF only 
| .edu only 

---

**Interactive relationship** (e.g. stimulation, inhibition, etc)

**Neutral relationship**- In already sensitized animals, blocking OX40  [ **TNFRSF4** ]  OX40L  [ **TNFSF4** ]  interactions at the time of inhalation of aerosolized antigen suppressed memory effector accumulation in lung draining lymph nodes and lung, and prevented eosinophilia, airway hyperreactivity, mucus secretion, and Th2 cyto kine production.  Ref: 12860930 J Exp MedJ Exp Med,
- Thus, OX40  [ **TNFRSF4** ]  OX40L  [ **TNFSF4** ]  interactions are pivotal to the efficiency of recall responses regulated by memory Th2 cells.  Ref: 12860930 J Exp MedJ Exp Med,
- Interaction of OX40  [ **TNFRSF4** ]  CD134  [ **TNFRSF4** ]  and its ligand, OX40L  [ **TNFSF4** ] , has been implicated in T cell activation and cell migration.  Ref: 12794142 J Immunol, 2003
- These results suggest that the OX40  [ **TNFRSF4** ]  OX40L  [ **TNFSF4** ]  system is crucial for induction of the allogeneic T cell response and the OX40  [ **TNFRSF4** ]  OX40L  [ **TNFSF4** ]  system is subsequent to and dependent on CD28 signalling, but is crucial for the end outcome of the human alloreactive T cell response.  Ref: 12757617 Immunology, 2003

**Non-interactive relationship** (e.g. studied together, co-existance, homology, etc.)

- In this study, we show that engagement of OX40  [ **TNFRSF4** ]  by an OX40L  [ **TNFSF4** ]  Ig fusion protein drives IFN gamma production by CD4 T cells and reduces eosinophilia and C. neoformans burden in the lung.  Ref: 12794142 J Immunol, 2003
- To further define the contribution of the OX40  [ **TNFRSF4** ]  OX40L  [ **TNFSF4** ]  system to the MLR, we set up a co culture system of CD4 T cells and allogeneic monocyte derived dendritic cells DCs.  Ref: 12757617 Immunology, 2003
- We first demonstrated that anti OX40  [ **TNFRSF4** ]  ligand  [ **TNFSF4** ]  anti OX40L  [ **TNFSF4** ]  monoclonal antibody mAb could markedly suppress the mixed leucocyte reaction MLR of peripheral blood mononuclear cells PBMC.  Ref: 12757617 Immunology, 2003
